# Antiepileptic Drug of Levetiracetam Decreases Centrotemporal Spike-Associated Activation in Rolandic Epilepsy

**DOI:** 10.3389/fnins.2018.00796

**Published:** 2018-11-27

**Authors:** Qirui Zhang, Fang Yang, Zheng Hu, Qiang Xu, Boris C. Bernhardt, Wei Quan, Qian Li, Zhiqiang Zhang, Guangming Lu

**Affiliations:** ^1^Department of Medical Imaging, Jinling Hospital, Southern Medical University, Nanjing, China; ^2^Department of Medical Imaging, Jinling Hospital, Nanjing University School of Medicine, Nanjing, China; ^3^Department of Neurology, Jinling Hospital, Nanjing University School of Medicine, Nanjing, China; ^4^Department of Neurology, Nanjing Children’s Hospital, Nanjing, China; ^5^Neuroimaging of Epilepsy Laboratory, McConnell Brain Imaging Centre, Montreal Neurological Institute and Hospital, McGill University, Montreal, QC, Canada

**Keywords:** Rolandic epilepsy, centrotemporal spike, antiepileptic drugs, EEG-fMRI, sequential HRFs analysis

## Abstract

The objective was to study the modulation effects of levetiracetam on the fMRI activation/deactivation patterns associated with centrotemporal spikes (CTS) in Rolandic epilepsy. Forty patients with Rolandic epilepsy, including levetiracetam-medicated patients (*n* = 20) and drug-naive patients (*n* = 20), were studied. Single and sequential hemodynamic response functions-based EEG-fMRI analysis was performed to detect dynamic activation/deactivation associated with CTS. Comparisons of spatiotemporal features of activation/deactivation were performed between the two groups. Both the groups (CTS were detected in 12 cases of levetiracetam-medicated group, and 11 cases of drug-naive group) showed CTS-associated activation in the Rolandic cortex, whereas activation strength, time-to-peak delay, and overall activation were diminished in the levetiracetam-medicated group. Moreover, the drug-naive group showed deactivation in the regions engaged in higher cognition networks compared with the levetiracetam-medicated group. Levetiracetam inhibits CTS-associated activation intensity and alters the temporal pattern of this activation in the epileptogenic regions, and it also affects the brain deactivation related to higher cognition networks. The findings sheds a light on the pharmocological mechanism of levetiracetam therapy on Rolandic epilepsy.

## Introduction

Rolandic epilepsy, also known as benign childhood epilepsy with centrotemporal spikes (CTS), is the most common form of idiopathic epilepsy syndrome in children (Beaussart, 1972). It is characterized by mild seizure symptoms and a high-voltage CTS on electroencephalography (EEG). Although Rolandic epilepsy was considered a benign epilepsy with favorable outcomes, the patients have been found to be associated with cognitive difficulties (motor control, visual learning, and attention) and language delays (reading and phonological processing) (Beaussart, 1972; Stephani, 2000; Giordani et al., 2006). Thus, rational use of antiepileptic drugs (AEDs) is recommended for seizure control and cognitive impairment relief of the child (Kossoff et al., 2007; von Stulpnagel et al., 2010).

Levetiracetam (LEV) is a relatively new and first-line AED drug in Rolandic epilepsy (Glauser et al., 2006, 2013; Mellish et al., 2015) due to its favorable characteristics on seizure control, tolerance, and pharmacokinetics (Schiemann-Delgado et al., 2012; Beaulieu, 2013). It functions by decreasing the released level of excitatory neurotransmitter through synaptic vesicle protein 2A (Crowder et al., 1999; Lynch et al., 2004; Crevecoeur et al., 2013). In addition, LEV is considered to have an enhancement effect on cognitive performance in epilepsy (Kossoff et al., 2007; von Stulpnagel et al., 2010; Cho et al., 2012; Wandschneider et al., 2014) and other states like mild cognitive impairment patients or healthy individuals (Bakker et al., 2015; Magalhaes et al., 2015). In the previous study, resting-state fMRI investigated long-term effect of LEV on brain activity in epileptogenic regions in Rolandic epilepsy (Zhang et al., 2017).

Although task fMRI (Wandschneider et al., 2014; Bakker et al., 2015) and resting-state fMRI (Zhang et al., 2017) revealed long-term effects of LEV on brain functions, these fMRI strategies cannot directly observe the instantaneous effect of LEV on the epileptic discharge-associated brain activation. In contrast simultaneous EEG and fMRI (EEG-fMRI) allows directly detecting blood oxygen level-dependent (BOLD) activation associated with interictal epileptiform discharges (Moeller et al., 2013). Using the dynamic analysis in combination, sequential hemodynamic response functions (HRFs)-based general linear model (GLM) analysis allowed observing the dynamic temporal characteristics of activation/deactivation associated with epileptic discharges (Bagshaw et al., 2004; Hawco et al., 2007; Jacobs et al., 2008; Moeller et al., 2008; Masterton et al., 2010; Benuzzi et al., 2012). It would also be more sensitive for detecting epileptic brain activation (Moeller et al., 2013).

In this study, we employed simultaneous EEG-fMRI and sequential HRFs analysis to investigate the alterations of the spatiotemporal features of CTS-associated activation/deactivation in Rolandic epilepsy, and further compared the difference between LEV-medicated patients and drug-naive patients. We hypothesized that LEV, as an antiepileptic drug, would inhibit the intensity and alter the temporal characteristics of CTS-associated activation in the epileptogenic regions of Rolandic epilepsy. This study would provide initial data for evaluating the instantaneous effect of AED on spike-associated activation/deactivation, and contribute to better understanding of pharmocological mechanism of LEV therapy on Rolandic epilepsy.

## Materials and Methods

### Participants

We studied forty patients with Rolandic epilepsy, recruited from Jinling Hospital between 2014.01 and 2017.01. The patients were diagnosed with Rolandic epilepsy with CTS according to the International League Against Epilepsy (ILAE) classification (Berg et al., 2010). The exclusion criteria were as follows: (1) younger than 5 years old, (2) focal abnormality on routine structural MRI, (3) no recording of CTS during EEG-fMRI. The details of the demographics and clinical data of all the patients are as follows: 23 boys and 17 girls; age: 8.8 ± 2.2 years; epilepsy duration: 17.7 ± 17.5 months; seizure frequency: 0.46 ± 0.46 time per month. Among these patients, 20 received monotherapy (*n* = 9) of LEV or polytherapy (*n* = 11) combining other AEDs (oxcarbazepine or lamotrigine), and the other 20 were not medicated by any AED at any time in the study.

This research was approved by the medical ethics committee in Jinling Hospital, Nanjing University School of Medicine, and written informed consent was obtained from guardian of each patient.

### Data Acquisition

Imaging was carried out in Jinling Hospital using an EEG-fMRI setup in a 3T MRI scanner (Siemens TimTrio, Erlangen, Germany) with a 32-channel head coil as well as an MRI-compatible EEG system (Brain Product, Munich, Germany). The patients were instructed to keep their eyes closed and remain awake during our study. Foam padding was placed between the head and the coil to minimize head movements. For EEG recordings, the electrode FCz was set as the reference and electrocardiography was recorded using an electrode placed on the back. Data were transmitted via an optic fiber cable from the amplifier placed inside the scanner room to a computer outside the scanner room with a sampling rate of 5,000 Hz. Functional MRI data were acquired using a T2^∗^-weighted single-shot echo planar imaging sequence (repetition time = 2000 ms, echo time = 30 ms, flip angle = 90 degrees). Thirty transverse slices (field of view = 240 × 240 mm^2^, in-plane matrix = 64 × 64, slice thickness = 4 mm, interslice gap = 0.4 mm, voxel size = 3.75 × 3.7 × 4 mm^3^), aligned along the anterior commissure–posterior commissure line, were acquired. In each session, a total of 500 volumes were collected, resulting in a total scan time of 1000 s. For each patient, 2–3 sessions were acquired. Subsequently, 3D T1-weighted anatomical images (repetition time = 2300 ms, echo time = 2.98 ms, flip angle = 90 degree, field of view = 256 × 256 mm^2^ and slice thickness = 1 mm) were obtained as structural reference (Liao et al., 2014). Other clinical diagnosis sequences (2D-T2WI, 2D-DWI, and 2D-FLAIR) were also acquired, but the findings are not presented here.

### Data Processing

**FIGURE 1:**
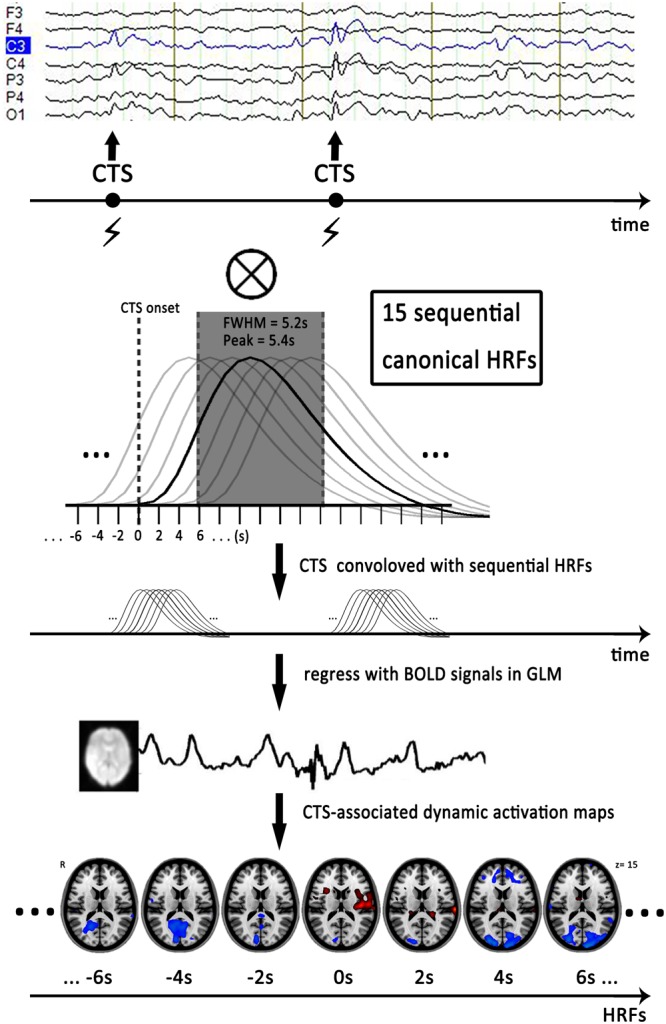
Flow chart of dynamic EEG-fMRI analysis. First, we marked time of centrotemporal spikes (CTS) onset on EEG by electroencephalographers. Second, we generated sequential HRFs consisting of 15 successive gamma functions of full-width half-maximum of 5.2 s, peak = 5.4 s and spaced 2 s between one another to model the dynamic BOLD changes induced by CTS. Finally, we convolved a boxcar function expressing the timing of CTS with sequential HRFs shifted from 14 s before to 14 s after the CTS (time point -14 to 14 s), to generate a series of regressors that modeled the dynamic activation/deactivation in individual level. Horizontal axis represented start time of the sequential HRFs. Actually, analysis with HRF centered at zero point (time point 0 s) was the conventional EEG-fMRI result with single HRF. The EEG and activation maps are from one of the patients with drug-naive. CTS, centrotemporal spikes; EEG, electroencephalography; HRF, hemodynamic response function; FWHM, full-width half-maximum; GLM, general-liner-model.

The simultaneous EEG data were offline-processed to remove gradient and ballistocardiogram artifacts using software Analyze 2.0 (Brain Products, Munich, Germany). An experienced electroencephalographer (Q.L.) and a neurologist (F.Y.) marked the CTS according to spatial distribution and morphology independently (Figure 1). Typically, CTS are mainly localized in the C3 and C4 (high central) or C5 and C6 (low central) supra-sylvian, and high-voltage spikes followed by slow waves are observed. The earliest peak of each CTS was marked as an event for subsequent analysis when polyspikes or repetitive spikes occurred. Disagreements were resolved and consensus was achieved via discussion. An fMRI subsession of 250 volumes was extracted from each patient under the guidance of EEG output. This fMRI subsession covered the maximal CTS number during the sessions. Patients who had no CTS recording during EEG-fMRI were excluded from the data analysis.

The fMRI data preprocessing was performed using SPM8 (Statistic Parameter Mapping^1^). After slice-timing adjustment and realignment for temporal differences and head motion correction, we coregistered individual 3D T1-weighted anatomical image to functional images. The 3D T1-weighted anatomical images were segmented (gray matter, white matter, and cerebrospinal fluid). Then, a non-linear spatial deformation was calculated from the gray matter images to a gray matter template in Montreal Neurological Institute (MNI) space. This transformation was then applied to the functional images, which were resliced at a resolution of 3 × 3 × 3 mm^3^. Data were spatially smoothed with an 8-mm full-width half-maximum isotropic Gaussian kernel. Six head-motion parameters were removed from the data by multiple linear regressions. Patients with head motion > 1.5 mm or 1.5° would be excluded from further analysis.

### Single and Sequential HRFs-Based GLM Analysis

To detect dynamic CTS-associated activation/deactivation, we first established a sequence of HRFs to model the dynamic BOLD changes associated with CTS. The sequential HRFs included 15 successive canonical HRF in SPM8 (full-width half-maximum of 5.2 s, peak = 5.4 s) and space 2 s between one another. At the individual level, we convolved a boxcar function expressing the timing of CTS with sequential HRFs shifted from 14 s before to 14 s after the CTS (time point -14–14s), to generate a series of regressors that modeled the dynamic activation/deactivation (Moeller et al., 2008; Zhang et al., 2014). Analysis with an HRF centered at the 0s time point corresponds to the single GLM-based EEG-fMRI result (Figure 1).

### Group Comparisons Between LEV-Medicated Group and Drug-Naive Group

To observe the spatial distributing patterns of CTS-associated activation in the two groups, the one-sample *t*-tests were calculated by individual contrast map of each group with specific HRF based on GLM in SPM8 separately. This analysis produced 15 t-maps representing CTS-associated BOLD activation modeled with different HRFs in each group. The statistical threshold was set at *p* < 0.01 (voxel-level *p* < 0.01 and cluster-level *p* < 0.05 with GRF correction).

To observe the differences of CTS-associated activation between the LEV-medicated group and the drug-naive group, temporal features on cluster level were studied further. On the basis of the one-sample *t*-test results of all time points, we selected region of interests (ROIs) from significant activation and deactivation clusters. The bilateral Rolandic cortex, posterior cingulate cortex (PCC), bilateral dorsolateral prefrontal cortex (dlPFC), and bilateral intraparietal sulcus (IPS) were selected in the drug-naive group whereas the left Rolandic cortex was selected in the LEV-medicated group. The activation/deactivation intensity within these ROIs was drawn from the contrast maps of each patient group at each time point, respectively. Then we calculated the group difference of dynamic activation/deactivation intensity at each time point using two-sample *t*-tests. Furthermore, the potential group difference between the monotherapy and polytherapy LEV-medicated patients was also investigated in the left Rolandic cortex (ROI from the LEV-medicated group). The statistical threshold was set at *p* < 0.05, and corrected for age, gender, epilepsy duration, seizure frequency, and CTS numbers during EEG-fMRI.

## Results

Among the 40 patients, 2 cases were excluded because the head motions were beyond 1.5 mm or 1.5°. In the remaining 38 patients, CTS were observed on 23 patients (12 of the LEV-medicated group, 11 of the drug-naive group). There were no significant differences in age, gender, seizure frequency before AED, the number of CTS during EEG-fMRI recordings, as well as head motion between both the two groups (*p* > 0.05). As expected, epilepsy duration and overall seizure frequency were longer and lower in the LEV-medicated group, respectively. The detail information is in Table 1. Furthermore, we tested the potential correlation between CTS-associated activation and covariates. There was no significant correlation of activation in bilateral Rolandic cortices (ROI from the drug-naive group) with LEV medication duration (LEV-medicated group: r = 0.13, *p* = 0.68), with epilepsy duration (drug-naive group: *r* = -0.17, *p* = 0.62; LEV-medicated group: *r* = -0.05, *p* = 0.88), with seizure frequency (drug-naive group: *r* = -0.36, *p* = 0.28; LEV-medicated group: *r* = 0.13, *p* = 0.68), or with CTS numbers during EEG-fMRI (drug-naive group: *r* = -0.30, *p* = 0.38; LEV-medicated group: *r* = 0.17, *p* = 0.60)in both the groups, respectively.

**Table 1:** Demographics and clinical data.

Protocols	LEV (*n* = 12)	Drug-naive (*n* = 11)	*p* value
Sex (M/F)	5/7	5/6	0.86
Age (mean ± SD), years	9.41 ± 2.39	7.63 ± 1.74	0.56
Epilepsy duration (months)	25.83 ± 13.11	2.54 ± 2.38	0.00^∗∗^
Overall seizure frequency (time/month)	0.19 ± 0.25	0.89 ± 0.048	0.00^∗∗^
Seizure frequency before AED (time/month)	0.66 ± 0.60	0.89 ± 0.048	0.68
CTS during EEG-fMRI	47.16 ± 41.41	90.63 ± 78.26	0.11
LEV medication duration (months)	20.91 ± 15.17		
LEV dosage (g/day)	1.00 ± 0.90		
Polytherapy	50%		
Head motion (mean FD)	0.20 ± 0.002	0.22 ± 0.006	0.31

*^∗∗^p < 0.01. AED, anti-epileptic drugs; LEV, levetiracetam; CTS, centrotemporal spikes; SD, standard deviation; FD, framewise displacement.*

### Single HRF-Based GLM Analysis

**FIGURE 2:**
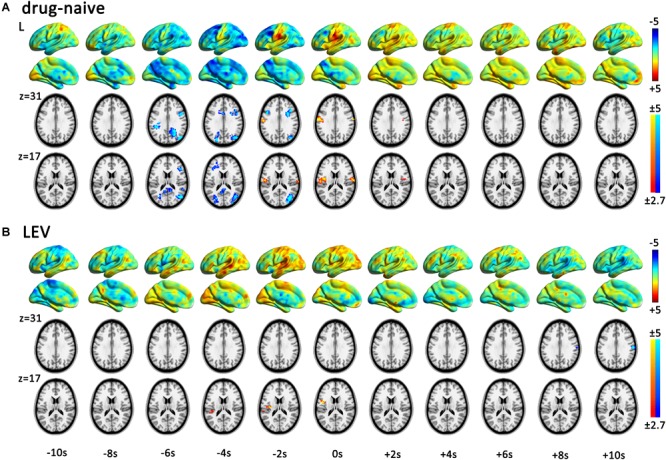
Spatial patterns of dynamic CTS-associated activation/deactivation in the drug-naive and LEV-medicated patients. The horizontal axis represented start time of the sequential HRFs. One-sample *t*-test within each group was performed in each time point in sequential hemodynamic response functions (HRF) analysis (time point -10 to 10 s) for drug-naive and levetiracetam (LEV)-medicated patients. Render maps with *p* = 1 in the left brain show the consecutively altered dynamic BOLD activity pattern. Slices maps with *p* < 0.01 (voxel-level *p* < 0.01, and cluster-level *p* < 0.05 with GRF correction) show significant activation and deactivation regions in each time point. Both the groups presented centrotemporal spikes (CTS)-associated activation in the Rolandic cortex. Viewed from the dynamic activation/deactivation, **(A)** drug-naive patients show Rolandic cortex activation during period from -2 to +2 s and extra Rolandic cortices deactivation before CTS onset (-6 to -2 s). **(B)** LEV-medicated patients show left Rolandic cortex activation during period from -4 to 0 s followed by deactivation after +8 s. LEV, levetiracetam.

**FIGURE 3:**
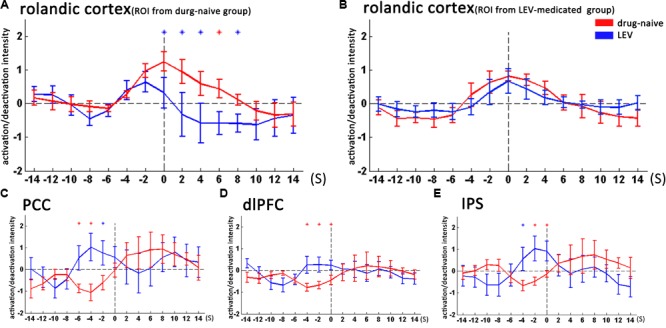
Temporal features of dynamic activation/deactivation associated with CTS in the Rolandic cortex and other brain regions in the drug-naive and LEV-medicated patients. Activation/deactivation changes during CTS from ROIs of the bilateral Rolandic cortex, left Rolandic cortex, posterior cingulate cortex (PCC), bilateral dorsolateral prefrontal cortex (dlPFC), and bilateral intraparietal sulcus (IPS). Mean value and standard error of activation/deactivation intensity time course in drug-naive patients and levetiracetam (LEV)-medicated patients visualize with red and blue lines, respectively. **(A)** In the bilateral Rolandic cortices (ROI from the drug-naive group), the LEV-medicated group showed earlier time-to-peak (time point -2 s) compared with the drug-naive group (time point 0 s). Moreover, the LEV-medicated group showed lower peak intensity and relatively rapid decline, resulting in lower activation intensity from 0 to +8 s overall (*p* < 0.05, with no correction). **(B)** In the left Rolandic cortex (ROI from the LEV-medicated group), there was no significant intensity difference between the two groups **(C–E)**. In the PCC, dlPFC, and IPS, the LEV group showed higher activation intensity compared with the drug-naive group before the CTS onset from -6 to -2 s, -4 to 0 s, -4 to 0 s, respectively. (*p* < 0.05, with no correction). ^∗^*p* < 0.05, ^∗^*p* < 0.01. LEV, levetiracetam; PCC, posterior cingulate cortex; dlPFC, dorsolateral prefrontal cortex; IPS, intraparietal sulcus.

fMRI analysis with single HRF (time point 0 s) revealed that CTS-associated activation was detected in the bilateral Rolandic cortices of the drug-naive group, whereas it was detected only in the left Rolandic cortex of the LEV-medicated group (Figure 2 and Table 2). A cluster level group comparison revealed that the LEV-medicated group had decreased activation compared with the drug-naive group in the Rolandic cortex of ROI from the drug-naive group. And there was no significant difference of activation intensity in the Rolandic cortex, ROI from the LEV-medicated group, between the two groups. (Figure 3).

**Table 2:** One-sample *t*-test of CTS-associated activation of single HRF in drug-naive and LEV-medicated patients.

	MNI coord			Voxels	Peak
	x	y	z		t value
***Drug-naive***		
Rolandic cortex _L	–63	–12	30	422	6.06
Rolandic cortex _R	69	–12	27	277	4.93
***LEV***		
Rolandic cortex _L	–45	–3	15	188	4.93

*L, left; R, right; LEV, levetiracetam; MNI, montreal neurological institute.*

### Sequential HRF-Based GLM Analysis

According to the dynamic activation/deactivation in sequential HRFs analysis, the LEV-medicated group had earlier beginning of CTS-associated activation (-4 to 0 s around CTS onset) in the Rolandic cortex than that in the drug-naive group (-2 to +2 s). The LEV-medicated group further showed deactivation at the 8th second after the CTS onset in the right Rolandic cortex. Deactivation in extra-Rolandic regions was also detected. In the drug-naive group, widely distributed cortical deactivation was found in regions consisting of PCC, bilateral dlPFC, and bilateral IPS during the period from -6 to -2 s (Figure 2).

**FIGURE 4:**
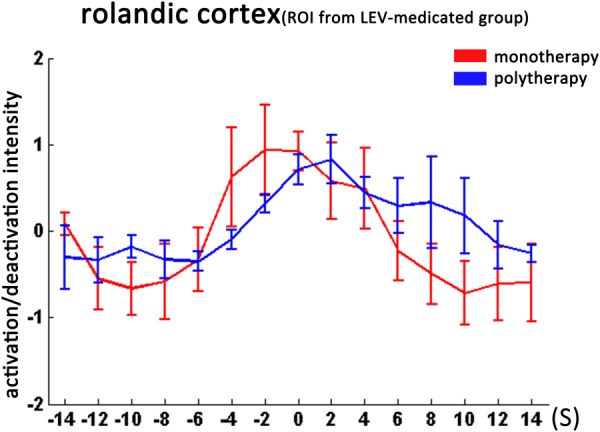
Temporal features of dynamic activation/deactivation associated with CTS in the Rolandic cortex in the monotherapy and polytherapy LEV-medicated patients. Activation/deactivation changes during CTS from ROI of left Rolandic cortex (ROI from LEV-medicated group). Mean value and standard error of activation/deactivation intensity time course in monotherapy LEV-medicated patients and polytherapy LEV-medicated patients visualize with red and blue lines, respectively. There was no significant intensity difference between the two groups.

We then studied the temporal evolution of CTS-associated activation of each patient group in cluster level. In the bilateral Rolandic cortices (ROI from the drug-naive group), the LEV-medicated group showed earlier time-to-peak (time point -2s) compared with the drug-naive group (time point 0 s). Moreover, the LEV-medicated group showed lower peak intensity and relatively rapid decline, resulting in lower activation intensity from 0 to +8 s overall. And there was no significant difference of activation intensity in the Rolandic cortex, ROI from the LEV-medicated group, between the two groups. However, there was no significant difference between the two groups in the Rolandic cortex of ROI from the LEV group. In extra-Rolandic regions, the LEV-medicated group had higher activation intensity compared with the drug-naive group before the CTS onset from -6 to -2 s, -4 to 0 s, -4 to 0 s in PCC, dlPFC and IPS, respectively (Figure 3). Furthermore, there was no significant difference of activation intensity in the Rolandic cortex between monotherapy and polytherapy LEV-medicated patients (Figure 4).

## Discussion

Using sequential HRFs-based EEG-fMRI analysis, we investigated the effect of LEV on dynamic CTS-associated activation/deactivation in Rolandic epilepsy patients. LEV medication decreased CTS-associated activation in the Rolandic cortex and altered temporal pattern of this activation which had earlier time-to-peak. Besides, LEV medication erased CTS-associated deactivation in the extra-Rolandic cortex regions which was related to higher cognition networks.

For the first time, this study demonstrated the LEV effect on CTS-associated activation/deactivation in Rolandic epilepsy. We found that LEV-medicated patients had decreased activation in the Rolandic cortex, i.e., the epileptogenic regions of Rolandic epilepsy (Kamada et al., 1998; Boor et al., 2007; Xiao et al., 2016; Zhang et al., 2017), compared with drug-naive patients. This result indicated that LEV could inhibit generation of epileptic activation. Converging clinical and EEG studies proved that LEV could reduce seizure frequency and suppress focal discharges in different childhood epilepsies (Capovilla et al., 2004; Glauser et al., 2006; von Stulpnagel et al., 2010; Bakke et al., 2011; Xiao et al., 2014). Previous task-fMRI studies revealed that LEV was associated with restoration of normal activation patterns in working memory paradigm (Wandschneider et al., 2014) and improvement in memory task performance and fMRI activation (Bakker et al., 2015) in temporal lobe epilepsy patients. Our previous work also exhibited that long-term antiepileptic effects of LEV could inhibit resting-state neural activity of Rolandic cortex in Rolandic epilepsy patients. The present study provided novel findings by directly observing the instantaneous effect of LEV on CTS-associated brain activation, which might contribute toward elucidating underlying antiepileptic pharmocological mechanisms of LEV (Wandschneider and Koepp, 2016).

We further compared the dynamic features of CTS-associated activation/deactivation between LEV-medicated patients and drug-naive patients, and found that LEV altered the temporal pattern of CTS-associated activation which had earlier time-to-peak. Sequential HRFs analysis allows observing the dynamic BOLD activation prior, post, and on the time point of epileptic discharges (Bagshaw et al., 2004; Hawco et al., 2007; Moeller et al., 2008; Benuzzi et al., 2012). We speculated that the usage of LEV might alter neuronal changes during CTS. Previous research also found similar shortened time-to-peak HRF in a group of Rolandic epilepsy patients mostly under AED medication, but they thought the alteration of HRF shape might result from the effect of Rolandic epilepsy (Masterton et al., 2010). Using the sequential-HRF strategy, our study delineated an entire picture of CTS-associated activation during a period around CTS onset free of constraint of possible specific HRF shape. The findings suggested that LEV can alter the temporal pattern of activation associated with CTS.

Furthermore, deactivation was detected in widely distributed extra-Rolandic cortices ahead of Rolandic cortex activation in drug-naive patients other than LEV-medicated patients. These cortices covered the regions commonly regarded as critical nodes in the higher cognitive networks (PCC for default network, dlPFC for executive control network, and IPS for dorsal attentional network). Patients with Rolandic epilepsy often show cognition and behavior abnormalities including motor control, visual learning, attention, and language dysfunction (Beaussart, 1972; Stephani, 2000; Giordani et al., 2006). The previous fMRI study also found network alteration in the default model, language, and attention (Lillywhite et al., 2009; Xiao et al., 2015, 2016). Notably, DMN were anticorrelation to Rolandic cortex in Rolandic epilepsy (Xiao et al., 2016), and the EEG-fMRI study found that epileptiform discharge affected cognition in various epilepsy by inhibiting the DMN. (Gotman et al., 2005; Ibrahim et al., 2014; Liao et al., 2014; Xiao et al., 2016). CTS frequency is also associated with cognition abnormalities in Rolandic epilepsy patients (Nicolai et al., 2006; Kim et al., 2014). Prospectively, neuropsychological data need further investigation to reveal association between cognition and higher cognitive network alterations induced by LEV.

Several methodological considerations and limitations are noteworthy. First, we used cross-sectional data from different patient groups, and did not obtain the data prior to starting the LEV medication. A longitudinal study directly comparing brain activity before and after medication will provide a more accurate result than that with cross-sectional data. Second, a relatively small cohort was included in this study, and a portion of patients underwent polytherapy of AEDs. Mixed AEDs medications might affect CTS-associated brain activation. In future study, employing a large number of patients without polytherapy would help in eliminating the effects of other drugs. Finally, lacking neuropsychological data constrained us to evaluate the effects of LEV on brain cognition.

## Conclusion

LEV inhibits CTS-associated activation intensity in the Rolandic cortex and alters the temporal pattern of this activation in the Rolandic cortex. LEV also affects the brain deactivation related to higher cognition networks. The findings shed lights on the pharmocological mechanism of LEV therapy on Rolandic epilepsy.

## Ethics Statement

This study was in accordance with the Declaration of Helsinki, and was approved by the ethics committee of Jinling Hospital. All participants provided written informed consent after a detailed description of this study.

## Author Contributions

QZ and ZZ recruited the subjects, collected the data, performed the analysis, generated the images, and wrote the manuscript. ZH and FY assisted with subjects recruitment and data collections. QL and FY worked as electroencephalographer for EEG diagnosis. WQ assisted with the data collection. QX assisted with the analysis of data. GL and BB contributed to the experimental design, revised the article, and developed the research concept.

## Conflict of Interest Statement

The authors declare that the research was conducted in the absence of any commercial or financial relationships that could be construed as a potential conflict of interest.

## Footnotes

1 http://www.fil.ion.ucl.ac.uk/spm
